# Tracking Down Duodenopancreatic Malignancy

**DOI:** 10.1155/2000/68578

**Published:** 2000-08

**Authors:** Constanze Samel-Westerhof, Stephan Samel, Walter Back, Jochen Gaa, Jörg Sturm

**Affiliations:** 1 Dept. of Gastroenterologie Universityhospital Mannheim Theodor-Kutzer-Ufer Mannheim D-68 135 Germany; 2 Dept. of Surgery Universityhospital Mannheim Theodor-Kutzer-Ufer Mannheim D-68 135 Germany; 3 Dept. of Pathology Universityhospital Mannheim Theodor-Kutzer-Ufer Mannheim D-68 135 Germany; 4 Dept. of Radiology Universityhospital Mannheim Theodor-Kutzer-Ufer Mannheim D-68 135 Germany

## Abstract

Background Malignant tumours of the duodenum are rare and often difficult to diagnose. Due to the small clinical experience with duodenal malignancies their prognosis is unknown and resection is the treatment of choice.

Case report Adding to a small series of incidental tumours, we report the case of a 65-year-old patient with primary extranodal (MALT-) lymphoma of the duodenum infiltrating the pancreatic head. The patient was admitted because of anaemia and epigastric discomfort with a history of Helicobacter- pylori associated gastric ulceration. Physical examination and bloodchemical values were otherwise normal. Endoscopy revealed duodenal ulceration but the biopsies taken from the ulceration did not give any evidence of malignancy or residual Helicobacter pylori infection. But MRT showed a circular intramural tumour of the duodenum. On laparotomy a large duodenal tumour adherent to the pancreatic head was found and a Whipple procedure was performed.

Conclusion Apart from describing the case of a rare lymphoproliferative disorder of the duodenum, this report illustrates the diagnostic difficulties with uncommon neoplasm's of the duodenopancreatic region and the value of MRT prior to resection of a duodenopancreatic mass.


*HPB Surgery*, 2000, Vol. 00, pp. 405-411
Reprints available directly from the publisher
Photocopying permitted by license only

© 2000 OPA (Overseas Publishers Association) N.V.
Published by license under
the Harwood Academic Publishers imprint,
part of The Gordon and Breach Publishing Group.
Printed in Malaysia.

### *Case Report*

### Tracking Down Duodenopancreatic Malignancy

CONSTANZE SAMEL-WESTERHOF[a], STEPHAN SAMEL[b,*], WALTER BACK[c], JOCHEN GAA[d] and JÖRG STURM[b]

[a]*Dept. of Gastroenterologie;* [b]*Dept. of Surgery;* [c]*Dept. of Pathology;* [d]*Dept. of Radiology, Universityhospital Mannheim, Theodor-Kutzer-Ufer, D-68 135 Mannheim, Germany*

*(Received 1 August 1999)*

**Background** **Malignant tumours of the duodenum are rare and often difficult to diagnose. Due to the small clinical experience with duodenal malignancies their prognosis is unknown and resection is the treatment of choice.**
**Case report** **Adding to a small series of incidental tumours, we report the case of a 65-year-old patient with primary extranodal (MALT-) lymphoma of the duodenum infiltrating the pancreatic head. The patient was admitted because of anaemia and epigastric discomfort with a history of Helicobacter-pylori associated gastric ulceration. Physical examination and bloodchemical values were otherwise normal. Endoscopy revealed duodenal ulceration but the biopsies taken from the ulceration did not give any evidence of malignancy or residual Helicobacter pylori infection. But MRT showed a circular intramural tumour of the duodenum. On laparotomy a large duodenal tumour adherent to the pancreatic head was found and a Whipple procedure was performed.**
**Conclusion** **Apart from describing the case of a rare lymphoproliferative disorder of the duodenum, this report illustrates the diagnostic difficulties with uncommon neoplasm's of the duodenopancreatic region and the value of MRT prior to resection of a duodenopancreatic mass.**

*Keywords:* MALT-Lymphoma, duodenum, pancreas, MRT

### INTRODUCTION

Malignant neoplasm's of the small bowel are rare and their prognosis is vague [1]. A recent epidemiological, US-American study [2] found an average annual incidence rate of 9.9 per million people for malignant tumours of the small intestines. The incidence was higher among men than among women and was found to be overall rising and rising with age. The most common histologic subtypes in this study were carcinoid tumours (3.8/million) and adenocarcinoma (3.7/million), sarcoma (1.3/million) and lymphoma (1.1/million). Smaller series have seen adenocarcinoma [3] or lymphoma [4] ahead in incidence. Adenocarcinoma seems to be more common in industrialised countries, where intestinal lymphomas are rather rare [5]. Intestinal lymphomas form a heterogeneous subgroup of malignant lymphomas currently classified in the *Kiel-*, or most recently in the *Revised European–American Classification of Lymphoid Neoplasm's* (=REAL) [6]. Intestinal B-Cell

*Corresponding author. Tel.: 49-621-3832225, Fax: 49-621-3833809, e-mail: stephan.samel@chir.ma.uni-heidelberg.de lymphoma, most often large cell lymphoma [7], is common in the ileum [5] but rare in the duodenum and may mimic adenocarcinoma of the pancreatic head or duodenum or other neoplasm's of the duodenopancreatic region [8].

Diagnosis of tumours of the duodenopancreatic region, as it has been experienced by various authors, is often obscured by vague abdominal symptoms *e.g.*, abdominal discomfort, nausea, vomiting or weight loss. Physical examination may reveal a palpable mass in patients with advanced tumour growth.

Endoscopic diagnosis of duodenal lesions often fails because of a cryptic tumour site or an essentially submucosal or intramural tumour growth as in the presented case [9–11]. Brush cytology may be of value in tumours of the papilla of Vater but often is not conclusive when taken from other sites [12]. Biopsy specimens, too, may fail to verify a histologic diagnosis due to endoscopically not accessible tumour sites. Laboratory examinations and contrast enhanced radiographs often lack characteristic findings as well.

Modern techniques of *all-in-one* MRT, MRCP and MRA may help to specify the diagnosis of tumours of the duodenopancreatic region [13] and to determine resectability [14]. Sensitivity of MRT is high but specificity varies with tumour site.

The case history, presented below, illustrates some of these diagnostic difficulties of characterising malignant tumours of the duodenopancreatic region and emphasises the value of high-quality MRT.

### CASE REPORT

A 65-year-old man was admitted because of anaemia, loss of weight, fatigue and epigastric discomfort with a suspected tumour of the duodenopancreatic region. *CT* of the abdomen, obtained prior to admission, had shown a mass in the duodenopancreatic region, suspicious for pancreatic adenocarcinoma.

On admission the patient was pale with a haemoglobin of 8.4 g/dl. Bloodchemical values, including serum electrophoresis, immune electrophoresis, differential blood count, and physical examination were normal.

The patient had a history of gastric ulceration and had received eradication for Helicobacter pylori infection one year prior to admission. He was under Phenobarbital medication for post-traumatic epilepsy but had not had seizures for 10 years. He denied recent fever, loss of weight, nausea or vomiting, jaundice, changes in bowel habits or quality of stool and any other major diseases. He had been operated for bilateral inguinal hernia but did not have other abdominal surgery.

On gastroduodenal *endoscopy* the gastroduodenal passage appeared to be normal. An ulcer was found in the descending duodenum proximal of the papilla. Mucosal biopsies from this site showed signs of chronic inflammatory ulceration without Helicobacter pylori infection.

An *endoscopic retrograde choledochopancreatography* was performed demonstrating a regular shape of bileduct and main pancreatic duct.

Contrary to the endoscopic findings, *hypotonic duodenography* demonstrated subtotal stenosis of the descending duodenum (Fig. 1).

MRT and MRCP were performed to further characterise the suspected tumour. *MRT* (Fig. 2) demonstrated a segmental circular wall thickening limited to the descending duodenum. A submucosal tumour, *e.g.*, lymphoma seemed to be the most likely diagnosis. The pancreatic head was normal in size. *MRCP* showed an unremarkable main pancreatic duct, excluding a pancreatic mass (Fig. 3).

*Endoscopy* was repeated and biopsies were taken from around the papilla of Vater. Histopathology of these biopsies again did not reveal any conclusive malignant findings.

On *laparotomy* a large duodenal tumour of circular intramural growth was found without

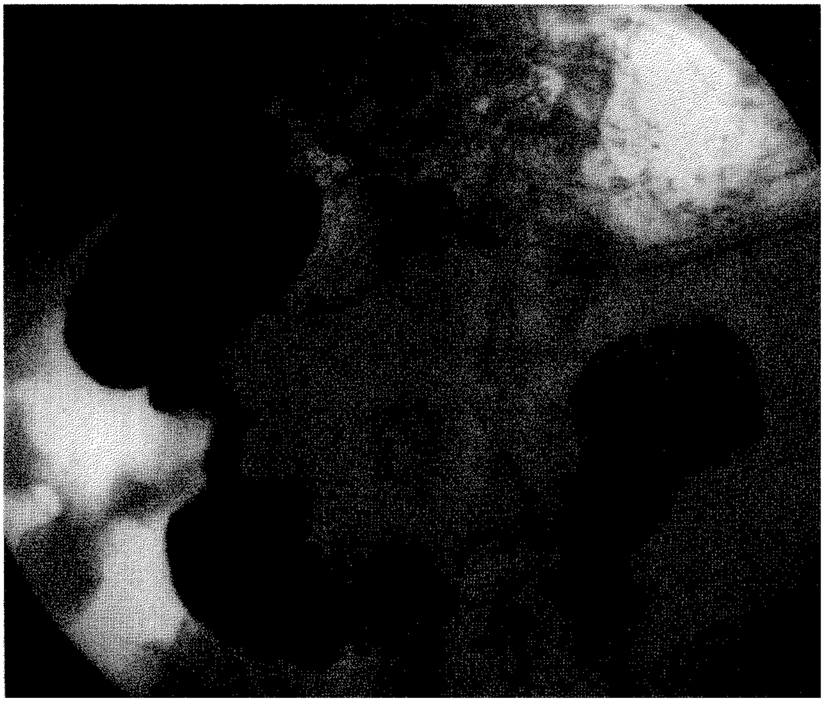

FIGURE 1 Hypotonic duodeno-radiography demonstrates a segmental stenosis of the descending duodenum.

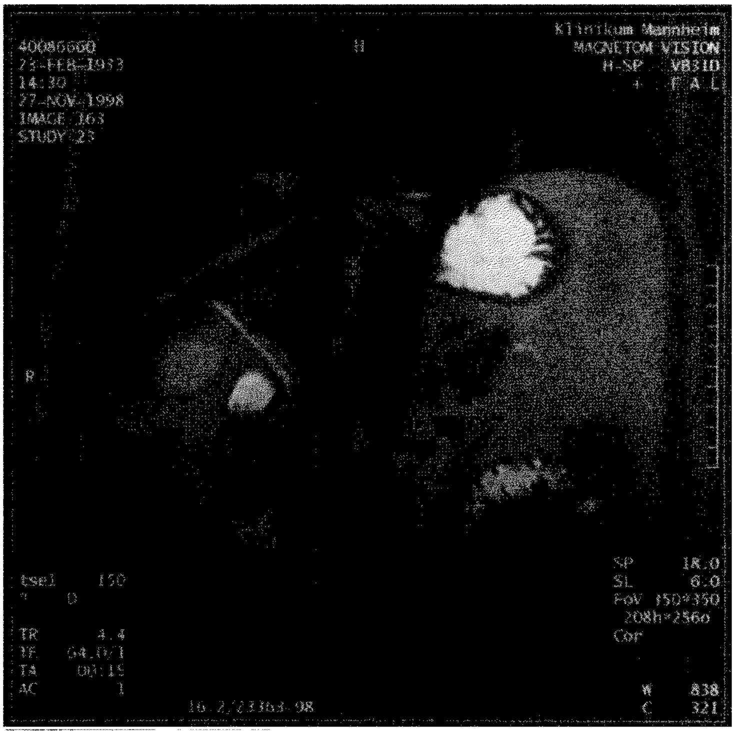

FIGURE 2 Longitudinal MRT scan of the upper abdomen showing a segmental circular wall thickening limited to the descending duodenum. The pancreatic head is normal in size.

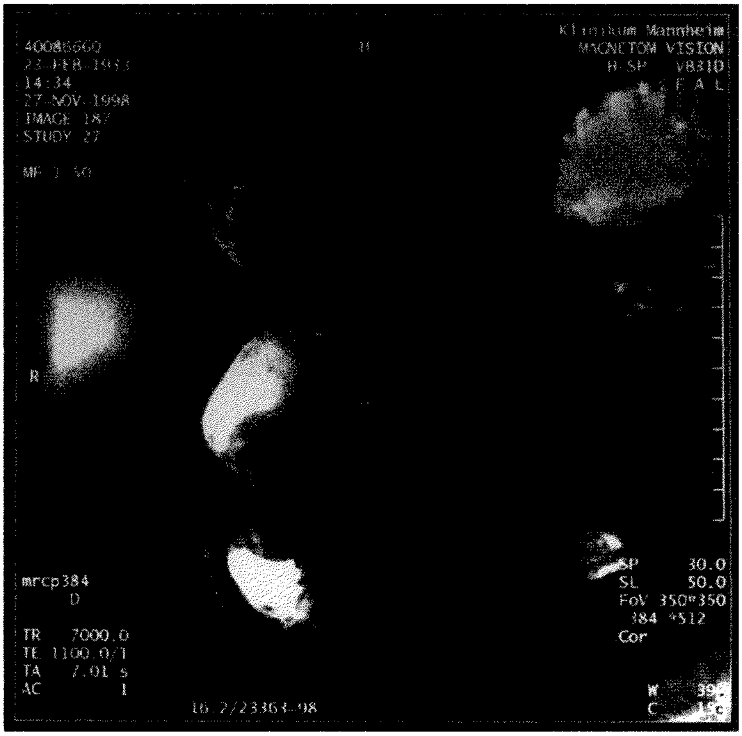

FIGURE 3 MRCP demonstrates an unremarkable main pancreatic duct, excluding a pancreatic mass.

apparent infiltration of mucosa or serosa. The tumour, however, was found to infiltrate the papilla of Vater and the pancreatic head. Suspecting malignancy a Whipple procedure was performed. The macroscopic inspection of the specimen revealed a semicircular infiltrative mass in the duodenal wall (Fig. 4). The patient recovered well and was discharged 10 days after surgery. Adjuvant radiotherapy was performed starting four weeks after surgery.

*Microscopic findings* The tumorous mass measured 5 cm in longitudinal length and was located in the wall of the duodenum. The duodenal mucosa overlying the mass showed flat ulceration. Microscopically there was a transmural infiltration of the entire thickness of the duodenal wall by proliferating lymphoid cell. These cells infiltrated the serosa and the pancreatic parenchyma also (Fig. 5a). The papilla of Vater was free of blastomatous infiltrates.

The diffuse lymphoid infiltrates contained a large population of CD-20 positive cells. CD-3 positive small T-cells were present in smaller numbers and were dispersed in the B-cell proliferates. Ki67-index was low and $\kappa$-lightchains were restricted (Fig. 5b) in favour of $\lambda$- lightchains (Fig. 5c). These findings are conclusive of a low grade B-cell lymphoma according to the Kiel-classification scheme. The infiltration of lymphoid cells with lymphofollicular hyperplasia in the duodenal mucosa next to the mucosal ulceration, which merges with the blastomatous infiltration of low grade B-cell lymphoma in the underlying deeper zones of the duodenal wall, is highly suggestive of a B-cell lymphoma derived from mucosa-associated lymphoid tissue (=MALT) of the duodenum. Helicobacter pylori, however, was not evident neither in the tumour specimen nor in the gastroduodenal biopsies taken preoperatively.

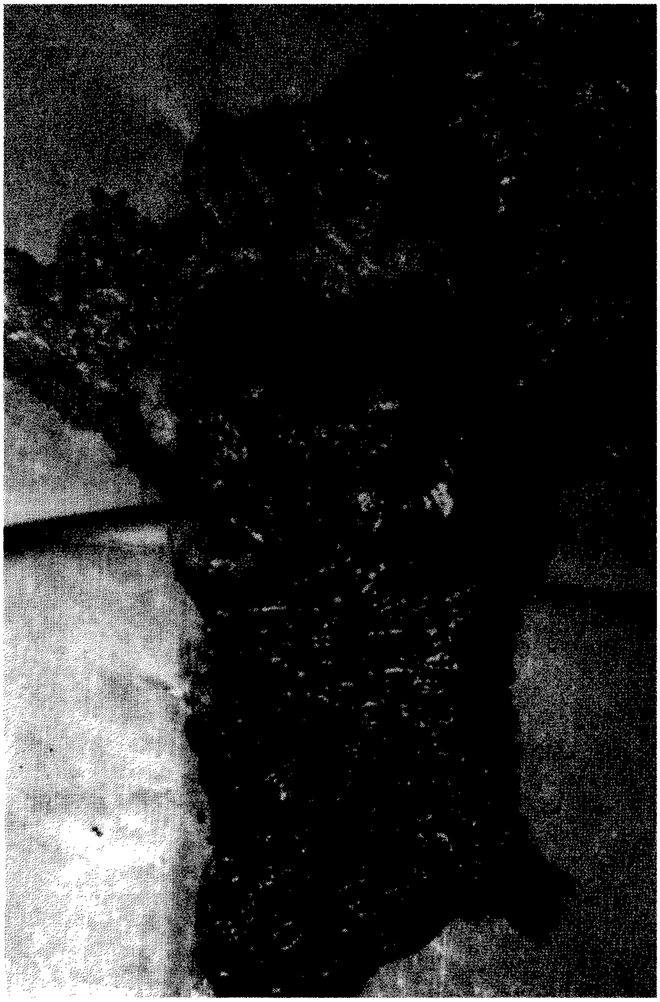

FIGURE 4 Gross photograph of the duodenum with a segmental intramural tumour of circular growth. The papilla of Vater is indicated by a probe.

### DISCUSSION

Reconsidering this case history, several questions arise: Could preoperative diagnosis been more effective, has surgery been necessary for this type of neoplastic lesion of duodenum and pancreas, and do primary (extranodal) duodenopancreatic lymphomas belong to the group of gastric lymphomas rather than to the group of intestinal lymphomas?

The few documented cases of primary (=extranodal) duodenal lymphoma reflect the variety of histologic subtypes of B-cell-lymphoma [15–17]. Lymphomas of the papilla of Vater may form another subgroup of intestinal B-cell-lymphoma [18–20]. Lymphoma of the stomach is known to occur in the setting of chronic Helicobacter pylori gastritis. Gastric lymphomas, located in the distal stomach, may invade the duodenum [21] and may mimic primary duodenal tumours there. In the presented case, duodenal ulceration and a history of Helicobacter pylori induced gastric ulceration may have been associated with the

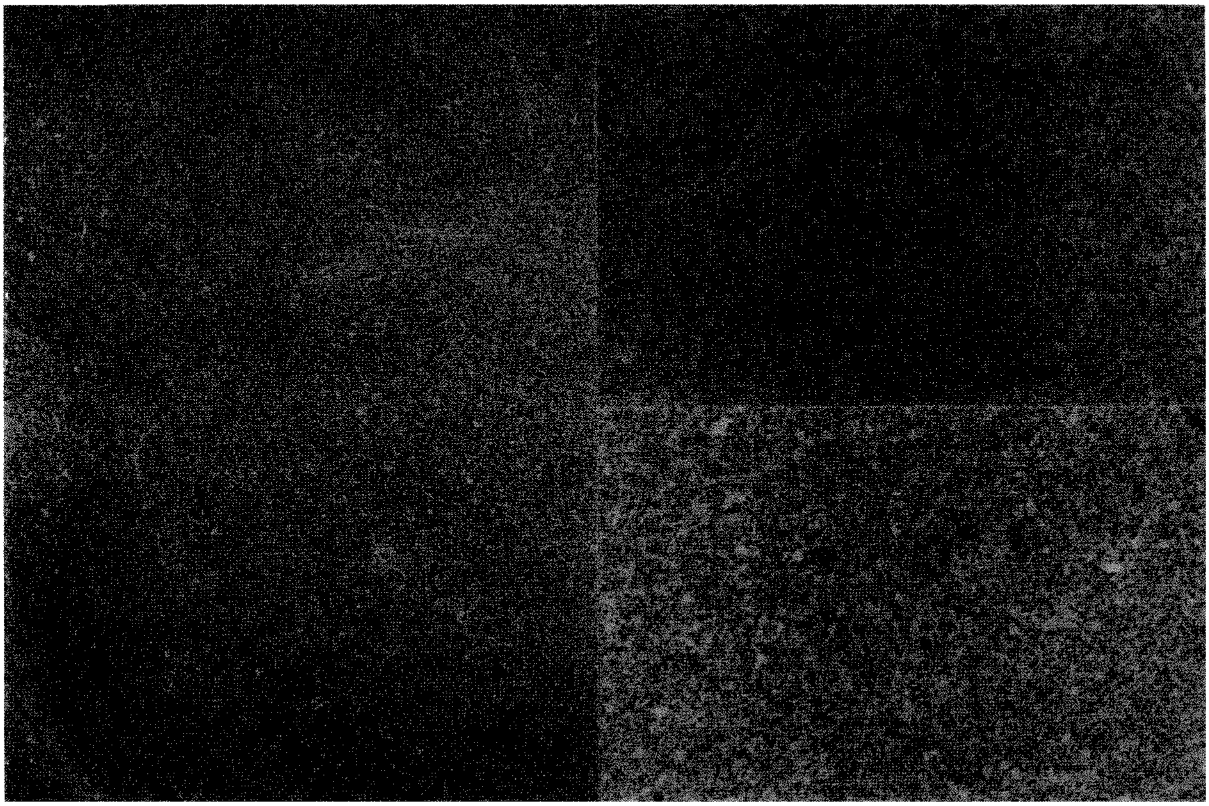

FIGURE 5 A photomicrograph of a low-power field of the tumour shows a diffuse proliferation of lymphoid cells with infiltration of the pancreatic parenchyma (left field). Medium-power fields of this lymphoma, immunostained for $\kappa$-lightchains (right upper field: negative) and for $\lambda$-lightchains (right lower field: positive in B-cells with lymphoplasmocytoid differentiation) showing a lightchain restriction as an evidence for a neoplastic proliferation.

pathogenesis of duodenal lymphoma. It has to remain unclear, however, whether the reported MALT-lymphoma represents a gastric lesion, which had skipped into the duodenum or a primary tumour.

Only little experience with the heterogeneous group of small bowel B-cell lymphomas has been gathered so far and treatment recommendations are still a matter of debate [22]. Several studies are currently on their way to compare radiotherapy, chemotherapy, surgery and multimodal approaches. There is only minor evidence [23], that eradication of Helicobacter pylori is beneficial in other than gastric lymphoma. Resection still is the predominant treatment for duodenopancreatic lymphoma [24, 25] as for any duodenopancreatic tumour of suspected malignancy, due to the lack of clinical data on medical treatment options, and, of course due to the difficulties of finding a preoperative diagnosis as well. It is another "dilemma" for surgeons [26] that the decision whether to resect or not to resect such a tumour often has to be made without a definite histopathologic diagnosis.

In the presented case, a tumour of the duodenopancreatic region was suspected but no histologic findings were obtained in the course of diagnostic procedures. Only MRT properly identified an intramural duodenal tumour. But experience in viewing such tumours still is too limited as to provide a specific characterisation of rare lesions. In such cases it will be left to the surgeon to decide, whether to resect a duodenopancreatic tumour of unknown histology or not.

### References

[1] Sanchez-Bueno, F., Garcia-Marcilla, J. A., Alonso, J. D., Acosta, J., Carrasco, L., Pinero, A. and Parrilla, P. (1998). Prognostic factors in primary gastrointestinal non-Hodgkin's lymphoma: a multivariate analysis of 76 cases. *Eur. J. Surg.*, **164**, 385–392.

[2] Chow, J. S., Chen, C. C., Ahsan, H. and Neugut, A. I. (1996). A population-based study of the incidence of malignant small bowel tumours: SEER, 1973–1990. *Int. J. Epidemiol.*, **25**, 722–728.

[3] Cunningham, J. D., Aleali, R., Aleali, M., Brower, S. T. and Aufses, A. H. (1997). Malignant small bowel neoplasms: histopathologic determinants of recurrence and survival. *Ann. Surg.*, **225**, 300–306.

[4] Garcia Marcilla, J. A., Sanchez Bueno, F., Aguilar, J. and Parrilla Paricio, P. (1994). Primary small bowel malignant tumors. *Eur. J. Surg. Oncol.*, **20**, 630–634.

[5] Neugut, A. I:, Jacobson, J. S., Suh, S. and Mukherjee, R. Arber (1998). The epidemiology of cancer of the small bowel. *Cancer Epidemiol. Biomarkers Prev.*, **7**, 243–251.

[6] Harris, N. L., Jaffe, E. S., Stein, H., Banks, P. M. and Chan, J. K. *et al.* (1994). A revised European–American classification of lymphoid neoplasms by the International Lymphoma Study Group. *Blood*, **84**, 1361–1392.

[7] Hansen, P. B., Vogt, K. C., Skov, R. L., Pedersen-Bjergaard, U., Jacobsen, M. and Ralfkiaer, E. (1998). Primary gastrointestinal non-Hodgkin's lymphoma in adults: a population-based clinical and histopathologic study. *J. Intern. Med.*, **244**, 71–78.

[8] Schoretsanitis, G., Livingstone, J. I., el-Japour, J. N., Watkins, N. and Wastell, C. (1994). Duodenal plasmacytoma: a rare extramedullary localization simulating carcinoma of the head of the pancreas. *Postgrad Med. J.*, **70**, 378–379.

[9] Chiu, K. W., Changchien, C. S., Chuah, S. K. and Chen, C. L. (1995). Endoscopic and image features in primary gastro-intestinal lymphoma: a 7-year experience. *Hepatogastroenterology*, **42**, 367–370.

[10] Wang, H. H., Lin, J. T., Chiu, C. C., Chiang, I. P., Wu, M. S. and Wang, T. H. (1995). Endoscopic features of mucosa-associated lymphoid tissue lymphoma of the duodenum. *Gastrointest Endosc.*, **41**, 258–61.

[11] Inai, M., Sakai, M., Kajiyama, T., Imada-Shirakata, Y., Kin, G., Inoue, K., Ueda, S. and Okuma, M. (1996). Endosonographic characterization of duodenal elevated lesions. *Gastrointest Endosc.*, **44**, 714–719.

[12] Bardales, R. H., Stanley, M. W., Simpson, D. D., Baker, S. J., Steele, C. T., Schaefer, R. F. and Powers, C. N. (1998). Diagnostic value of brush cytology in the diagnosis of duodenal, biliary, and ampullary neoplasms. *Am. J. Clin. Pathol.*, **109**, 540–548.

[13] Trede, M., Rumstadt, B., Wendl, K., Gaa, J., Tesdal, K., Lehmann, K. J., Meier-Willersen, H. J., Pescatore, P. and Schmoll, J. (1997). Ultrafast Magnetic Resonance Imaging Improves Staging of Pancreatic Tumours. *Ann. Surg.*, **226**, 393–407.

[14] McFarland, E. G., Kaufman, J. A., Saini, S., Halpern, E. F., Lu, D. S., Waltman, A. C. and Warshaw, A. L. (1996). Preoperative staging of cancer of the pancreas: value of MR angiography *versus* conventional angiography in detecting portal venous invasion. *Am. J. Roentgenol.*, **166**, 37–43.

[15] Muchmore, J. H., Haddad, C. G. and Goldwag, S. (1994). Primary non-Hodgkin's lymphoma of the duodenum. *Am. Surg.*, **60**, 924–928.

[16] Kawai, T., Tada, T., Yokoyama, Y., Joh, T. and Itoh, M. (1998). Lymphoma arising in mucosa-associated lymphoid tissue of the duodenal bulb. *J. Gastroenterol.*, **33**, 97–101.

[17] Yoshikawa, I., Murata, I., Tanaka, Y., Kanagawa, K., Tabaru, A., Itoh, H. and Otsuki, M. (1996). Case report: primary CD3O (Ki-1)-positive anaplastic large cell lymphoma of the duodenum. *Dig. Dis. Sci.*, **41**, 2343–2347.

[18] Misdraji, J., Fernandez del Castillo, C. and Ferry, J. A. (1997). Follicle center lymphoma of the ampulla of Vater presenting with jaundice: report of a case. *Am. J. Surg. Pathol.*, **21**, 484–488.

[19] Pawade, J., Lee, C. S., Ellis, D. W., Vellar, I. D. and Rode, J. (1994). Primary lymphoma of the ampulla of Vater. *Cancer*, **73**, 2083–2086.

[20] Zhu, L., Slee, G. R., Domenico, D. R., Mao, C. and Howard, J. M. (1995). B-cell lymphoma of ampulla of Vater: observation for six years. *Pancreas.*, **10**, 208–210.

[21] Cho, K. C., Baker, S. R., Alterman, D. D., Fusco, J. M. and Cho, S. (1996). Transpyloric spread of gastric tumours: comparison of adenocarcinoma and lymphoma. *Am. J. Roentgenol.*, **167**, 467–469.

[22] Banks, P. M. and Isaacson, P. G. (1999). MALT lymphomas in 1997. Where do we stand? *Am. J. Clin. Pathol.*, **111**, (1 Suppl. 1), 75–83.

[23] Nagashima, R., Takeda, H., Maeda, K. and Ohno, S. Takahashi (1996). Regression of duodenal mucosa-associated lymphoid tissue lymphoma after eradication of Helicobacter pylori. *Gastroenterology*, **111**, 1674–1678.

[24] Law, M. M., Williams, S. B. and Wong, J. H. (1996). Role of surgery in the management of primary lymphoma of the gastrointestinal tract. *J. Surg. Oncol.*, **61**, 199–204.

[25] Rumstadt, B., Wendl, K., Schuster, K. and Knoll, M. (1997). The Whipple pancreatoduodenectomy in the treatment of pancreatic lymphoma. *Pancreas.*, **15**, 211–213.

[26] Trede, M. (1987). Treatment of pancreatic carcinoma: the surgeons dilemma. *Br. J. Surg.*, **74**, 79–80.

